# Association of glycemic variability and the presence and severity of coronary artery disease in patients with type 2 diabetes

**DOI:** 10.1186/1475-2840-10-19

**Published:** 2011-02-25

**Authors:** Gong Su, Shuhua Mi, Hong Tao, Zhao Li, Hongxia Yang, Hong Zheng, Yun Zhou, Changsheng Ma

**Affiliations:** 1 Department of Cardiology, Beijing Anzhen Hospital of Capital Medical University, Beijing, China; 2 Beijing Emergency Center of Heart, Lung & Blood Vessel Diseases, Beijing, China

## Abstract

**Background:**

Glucose variability is one of components of the dysglycemia in diabetes and may play an important role in development of diabetic vascular complications. The objective of this study was to assess the relationship between glycemic variability determined by a continuous glucose monitoring (CGM) system and the presence and severity of coronary artery disease (CAD) in patients with type 2 diabetes mellitus (T2DM).

**Methods:**

In 344 T2DM patients with chest pain, coronary angiography revealed CAD (coronary stenosis ≥ 50% luminal diameter narrowing) in 252 patients and 92 patients without CAD. Gensini score was used to assess the severity of CAD. All participants' CGM parameters and biochemical characteristics were measured at baseline.

**Results:**

Diabetic patients with CAD were older, and more were male and cigarette smokers compared with the controls. Levels of the mean amplitude of glycemic excursions (MAGE) (3.7 ± 1.4 mmol/L vs. 3.2 ± 1.2 mmol/L, p < 0.001), postprandial glucose excursion (PPGE) (3.9 ± 1.6 mmol/L vs. 3.6 ± 1.4 mmol/L, p = 0.036), serum high-sensitive C-reactive protein (hs-CRP) (10.7 ± 12.4 mg/L vs. 5.8 ± 6.7 mg/L, p < 0.001) and creatinine (Cr) (87 ± 23 mmol/L vs. 77 ± 14 mmol/L, p < 0.001) were significantly higher in patients with CAD than in patients without CAD. Gensini score closely correlated with age, MAGE, PPGE, hemoglobin A_1c _(HbA_1c_), hs-CRP and total cholesterol (TC). Multivariate analysis indicated that age (p < 0.001), MAGE (p < 0.001), serum levels of HbA_1c _(p = 0.022) and hs-CRP (p = 0.005) were independent determinants for Gensini score. Logistic regression analysis revealed that MAGE ≥ 3.4 mmol/L was an independent predictor for CAD. The area under the receiver-operating characteristic curve for MAGE (0.618, p = 0.001) was superior to that for HbA_1c _(0.554, p = 0.129).

**Conclusions:**

The intraday glycemic variability is associated with the presence and severity of CAD in patients with T2DM. Effects of glycemic excursions on vascular complications should not be neglected in diabetes.

## Background

Cardiovascular diseases, including coronary artery disease (CAD), are the major causes of morbidity and cardiovascular death in patients with type 2 diabetes mellitus (T2DM) [1,2]. Diabetic patients usually present various factors contributing to the risk of cardiovascular diseases, which include hyperglycemia, fluctuation of blood glucose, central obesity, hyperlipidemia and hypertension and so on [2]. Glycemic disorders are important components of these risk factors.

Interventional studies have established that cardiovascular complications are mainly or partly dependent on sustained chronic hyperglycemia [3,4]. This glycemic disorder can be estimated as a whole from the determination of hemoglobin A_1c _(HbA_1c_) level, which integrates both basal and postprandial hyperglycemia [5,6]. The incidence of cardiovascular complications has been identified as depending on HbA_1c _and on fasting and/or postprandial hyperglycemia, whether these parameters were investigated concomitantly or separately [7,8]. However, the glycemic disorders in T2DM are not solely limited to sustained chronic hyperglycemia but can be extended to the glycemic variability that includes both upward and downward acute glucose changes [9]. Some studies have showed that fluctuations of glucose seem to have more deleterious effects than sustained hyperglycemia in the development of diabetic complications as acute glucose swings activate the oxidative stress [10,11]. Recent studies have indicated that glycemic variability might play a role in the pathogenesis of atherosclerosis and may be an independent risk factor for cardiovascular complications in diabetic patients [10-13]. Therefore, in order to assess the risk of diabetes, it may be necessary to evaluate not only the mean level of glycemic control, but also the extent of glucose excursions. However, there have been no sufficient studies presented so far that specifically evaluated the relationship between glycemic variability and coronary artery disease in diabetic patients.

In this study, we examined the parameters of glucose profile using continuous glucose monitoring system (CGMS) in T2DM patients with CAD, and established a correlation between glycemic variability and the severity of coronary artery disease assessed by coronary angiogram, using the Gensini score.

## Methods

### Study population

We studied consecutive T2DM patients with chest pain, who underwent coronary angiography at the Department of Cardiology, Beijing Anzhen Hospital of Capital Medical University. The inclusion criteria were: (i) referral to coronary angiography, due to chest pain; (ii) admission glucose < 16.7 mmol/L, and without diabetic ketosis or nonketotic hyperosmolar coma. Patients' anti-hyperglycemic therapy would be maintained as usual until CGMS monitoring completed. Otherwise, the patient would be excluded from the study. In addition, patients with acute coronary syndrome, infectious diseases, previous coronary artery bypass graft surgery and previous percutaneous coronary intervention were not included. Thus, 344 patients with complete data were included in the final analysis. Hypertension was defined as systolic blood pressure ≥ 140 mmHg and/or diastolic blood pressure ≥ 90 mmHg or treatment with oral anti-hypertension drugs. Hyperlipidemia was diagnosed according to guideline of the National Cholesterol Education Program (ATP III). T2DM was diagnosed according to the American Diabetes Association criteria [14]. Renal insufficiency was defined as estimated glomerular filtration rate (eGFR) < 60 (ml/min/1.73 m^2^). eGFR value was calculated by MDRD equation [15]. The study was approved beforehand by the Ethics Committee of Beijing Anzhen Hospital of Capital Medical University and the procedures followed were in accordance with the institutional guidelines. The study complied with the declaration of Helsinki and informed consent was obtained from all patients.

### Continuous glucose monitoring

All patients were equipped with CGMS (Medtronic MiniMed, USA), and were monitored for 72 consecutive hours after admission. A CGMS sensor was inserted into the subcutaneous abdominal fat tissue, calibrated according to the standard Medtronic MiniMed operating guidelines. During CGMS monitoring, patients checked their blood glucose level with a self-monitoring of blood glucose (SMBG) device (Medisafe Mini, Terumo, Japan) at least 4 times per day. Then, they entered the SMBG data and time of each meal into the CGMS. After monitoring for 72 hours, the recorded data were downloaded into a personal computer for analysis of the glucose profile and glucose excursion parameters with MiniMed Solutions software. Analysis was limited to the data obtained from the intermediate 48 hours of recording to avoid bias due to insertion and removal of the CGMS or insufficient stability of the monitoring system. Since measurable range of glucose by CGMS was mechanically limited from 2.2 to 22.2 mmol/L, the case showing the data out of this range was excluded from the study. After downloading the recorded data, three indices of glycemic variability were analyzed from the intermediate 48 hours of data [16]: the mean amplitude of glycemic excursions (MAGE), the mean of daily differences (MODD) and postprandial glucose excursion (PPGE). The MAGE was calculated by measuring the arithmetic mean of the differences between consecutive peaks and nadirs, provided that the differences are greater than one standard deviation of the mean glucose value. The MODD was calculated as the mean of the absolute differences between glucose values at the same time of two consecutive days. The PPGE was obtained by calculating the post-breakfast increments in blood glucose.

### Coronary Angiography

After CGMS monitoring finished, coronary artery angiography was performed by using standard Judkins techniques or a radial approach. During cardiac catheterization, nitroglycerine was administrated routinely in all cases suspected of having coronary spasm. Angiographic analysis was carried out by two experienced interventional cardiologists who were blinded to the study protocol. Angiography results were divided into CAD (≥50% obstruction in ≥1 coronary artery) group and non-CAD group. Gensini score assesses the severity of coronary artery disease: it grades narrowing of the lumen of the coronary artery and scores it as 1 for 1-25% narrowing, 2 for 26-50% narrowing, 4 for 51-75%, 8 for 76-90%, 16 for 91-99% and 32 for a completely occluded artery. This score is then multiplied by a factor according to the importance of the coronary artery. The multiplication factor for a left main stem (LMS) lesion is 5. It is 2.5 for proximal left anterior descending artery (LAD) and proximal circumflex artery (CX) lesions, 1.5 for a mid-LAD lesion, and 1 for distal LAD, mid/distal CX and right coronary artery lesions. The multiplication factor for any other branch is 0.5.

### Biochemical Investigations

Blood samples were collected after overnight fasting and stored at -70°C prior to analysis. Serum creatinine, fasting plasma glucose (FPG), high-sensitive C-reactive protein (hs-CRP), total cholesterol(TC), low-density lipoprotein-cholesterol (LDL-C), high-density lipoprotein-cholesterol (HDL-C) and triglyceride (TG) levels were measured by automatic biochemical analyzer (Hitachi 747, Tokyo, Japan). Serum concentration of hemoglobin A_1c _(HbA_1c_) was determined by high-performance liquid chromatographic method using automatic HbA_1c _analyzer (Tosoh HLC-723G7, Japan).

### Statistical Analysis

All statistical analyses were performed by using SPSS for Windows 13.0 (SPSS Inc, Chicago, IL, USA). Data are presented as frequencies and percentages for categorical variables and mean ± SD for continuous variables, unless otherwise indicated. Differences between two groups were assessed by using the Chi-square and unpaired t-tests. Correlation between continuous variables was determined by Pearson correlation coefficients. Binary logistic regression analysis was performed to identify the relative risk of the glycemic variability for the presence of CAD, expressed as odds ratios (OR) with 95% confidence intervals (CI). The predictive values of MAGE and HbA_1c _for the presence of CAD were calculated by constructing receiver-operating characteristic (ROC) curves. To ascertain the independent contribution to severity of CAD, multivariate stepwise linear regression analysis was made with Gensini score as the dependent variable control for FPG, duration of diabetes, blood pressure, age, MAGE, MODD, PPGE, HbA_1c_, BMI, hs-CRP and TC. A value of p < 0.05 was considered statistically significant.

## Results

### Clinical Characteristics

**Figure 1:**
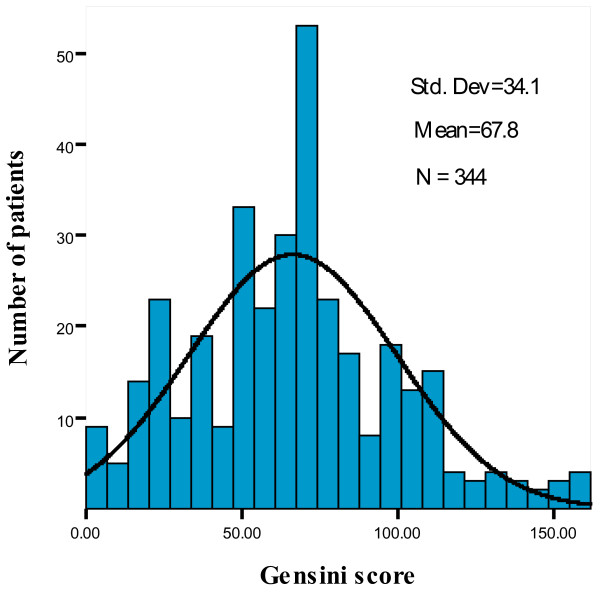
**Distribution of Gensini score among participants**.

Among the 344 participants, 252 patients had angiographically-proven CAD and 92 had almost normal coronary arteries (Non-CAD group). Baseline characteristics of the two groups are shown in Table 1. The Gensini score ranged from 0 to 162 with a mean of 67.8 ± 34.1, as shown in Figure 1. Diabetic patients with CAD were older, and more were male and cigarette smokers compared with the patients without CAD. The CAD group had significantly higher levels of hs-CRP and lower levels of eGFR, but no significant differences in blood pressure, hyperlipidemia, BMI, TC, HDL-C, LDL-C and TG levels. In addition, most of the diabetic patients in the CAD group and Non-CAD group were receiving statin medication (69.4% vs. 61.9%, p > 0.05), and did not differ significantly with the use of insulin (40.9% vs. 35.1%, p > 0.05). There was no difference in treatment with other antidiabetic agents in diabetic patients with and without CAD.

**Table 1:** Baseline characteristics of diabetic patients with (CAD) and without (Non-CAD) coronary artery disease, as defined by coronary angiography

	Non-CAD group (n = 92)	CAD group (n = 252)	p value
Male (%)	48 (52.2)	165 (65.5)	0.025
Age (years)	61 ± 9	65 ± 9	< 0.001
Cigarette smoking (%)	19 (20.7)	91 (36.1)	0.007
Hypertension (%)	56 (60.9)	177 (70.2)	NS
Blood pressure			
Systolic (mmHg)	123 ± 17	126 ± 20	NS
Diastolic (mmHg)	76 ± 8	78 ± 9	NS
Hyperlipidemia (%)	48 (52.2)	154 (61.1)	NS
Duration of diabetes (months)	58 ± 68	78 ± 77	0.022
Oral anti-hyperglycemic therapy (%)	42 (45.7)	111 (44.0)	NS
Insulin (%)	33 (35.9)	103 (40.9)	NS
Statins (%)	57 (61.9)	175 (69.4)	NS
Cholesterol			
Total cholesterol (mmol/L)	4.6 ± 1.1	4.8 ± 1.2	NS
LDL-C (mmol/L)	2.7 ± 0.8	2.9 ± 1.0	NS
HDL-C (mmol/L)	1.0 ± 0.2	1.1 ± 0.3	NS
Triglycerides (mmol/L)	2.1 ± 1.2	2.2 ± 1.6	NS
Creatinine (μmol/L)	77 ± 19	87 ± 23	< 0.001
eGFR (ml/min/1.73 m^2^)	87 ± 23	79 ± 18	< 0.001
Fasting plasma glucose (mmol/L)	7.3 ± 1.8	7.6 ± 2.4	NS
Hemoglobin A_1c _(%)	7.5 ± 1.4	7.6 ± 1.5	NS
MAGE (mmol/L)	3.2 ± 1.2	3.7 ± 1.4	< 0.001
MODD (mmol/L)	2.4 ± 1.0	2.5 ± 1.0	NS
PPGE (mmol/L)	3.6 ± 1.4	3.9 ± 1.6	0.036
BMI (kg/m^2^)	26.0 ± 2.2	26.4 ± 2.4	NS
hs-CRP (mg/L)	5.8 ± 6.7	10.7 ± 12.4	< 0.001

Abbreviations: CAD, coronary artery disease; LDL-C, lower-density lipoprotein-cholesterol; HDL-C, high-density lipoprotein-cholesterol; eGFR, estimated glomerular filtration rate; MAGE, the mean amplitude of glycemic excursions; MODD, the mean of daily differences; PPGE, postprandial blood glucose excursions; BMI, body mass index; hs-CRP, highsensitive-C reactive protein.

Data are mean ± SD and number (%).

### Glycemic variability, HbA_1c _and Fasting Plasma Glucose Levels

MAGE and PPGE were significantly higher in patients with CAD than in patients without CAD, but MODD did not significantly differ. CAD patients had longer duration of diabetes. There was also no significant difference in the HbA_1c _and FPG levels between two groups (Table 1).

### Relationship between Gensini Score and Glycemic variability, Age and Biochemical Parameters

**Figure 2:**
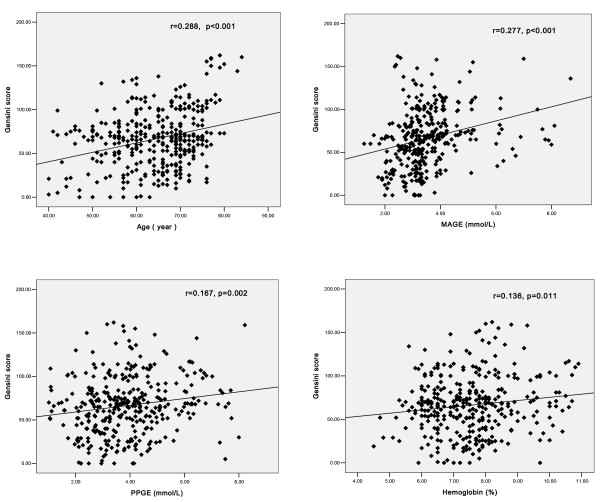
**Simple linear correlation of Gensini score and age, MAGE, PPGE and hemoglobin A_1c _in patients with type 2 diabetes**.

Pearson correlation analysis showed that Gensini score was closely related to MAGE(r = 0.277, p < 0.001), age (r = 0.288, p < 0.001), PPGE (r = 0.167, p = 0.002) and the level of HbA_1c _(r = 0.136, p = 0.011) (Figure 2), and correlated significantly with the levels of hs-CRP (r = 0.132, p = 0.014) and TC (r = 0.108, p = 0.045), but not with MODD, eGFR, duration of diabetes, blood pressure and other factors. In the final multivariate linear regression analysis model that explained 19.1% (adjusted multiple R^2 ^= 0.191) of the variation in Gensini score, the independent determinants were age (p < 0.001), MAGE (p < 0.001), hs-CRP (p = 0.002) and HbA_1c _(p = 0.022) (Table 2).

**Table 2:** Multivariate analysis of determinants of Gensini score

Independent variables	Unstandardized coefficients		Standardized coefficients β	t	p value
				
	B	SE			
Constant	-55.587	14.441		-3.849	0
Age	1.004	0.181	0.270	5.533	0.000
MAGE	7.010	1.466	0.237	4.783	0.000
hs-CRP	0.468	0.148	0.159	3.164	0.002
HbA_ 1c_	2.641	1.145	0.114	2.306	0.022
Adjusted multiple R ^2^	0.191				0

Abbreviations: MAGE, the mean amplitude of glycemic excursions; hs-CRP, highsensitive-C reactive protein; HbA_1c_, Hemoglobin A_1c._

### Logistic Regression Analysis for Independent Determinants of CAD

**Figure 3:**
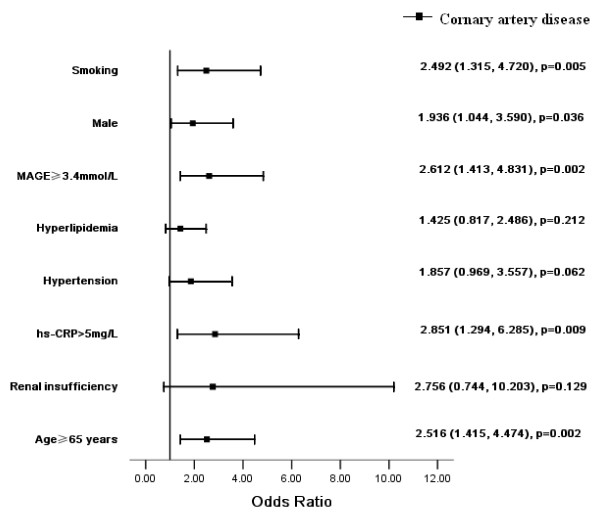
**Multivariate analysis for independent determinants of coronary artery disease (CAD)**. Smoking, male, older age, MAGE and hs-CRP were independent risk factors for CAD.

Cigarette smoking, male, older age (≥65 years), high MAGE level (≥3.4 mmol/L) and high hs-CRP level (> 5 mg/L) were found to be independent risk factors for the presence of CAD in T2DM patients, having OR 2.492 (p = 0.005), 1.936 (p = 0.036), 2.516 (p = 0.002), 2.612 (p = 0.002), and 2.851 (p = 0.009), respectively (Figure 3).

### ROC Curve for MAGE and HbA_1c _in Predicting CAD in T2DM

**Figure 4:**
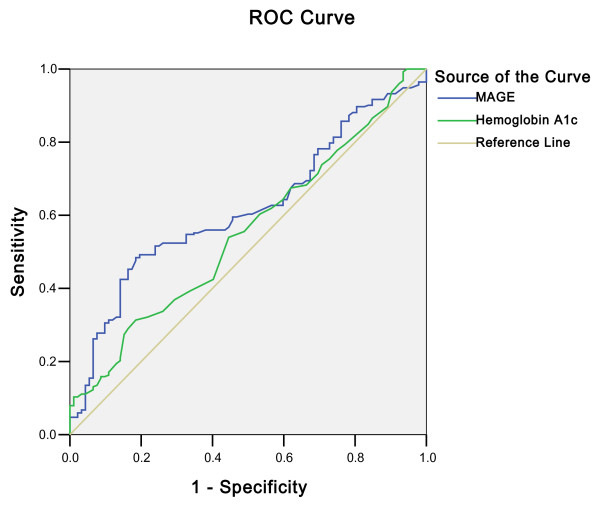
**Receiver-operating characteristic (ROC) curve for MAGE and hemoglobin A_1c _(HbA_1c_) in predicting coronary artery disease (CAD) in patients with type 2 diabetes (T2DM)**. Area under the receiver-operating characteristic curve: MAGE 0.618 (95% CI 0.555, 0.680), p = 0.001; HbA_1c _0.554 (95% CI 0.487, 0.620), p = 0.129. MAGE, but not HbA_1c_, displayed significant value in predicting CAD in patients with T2DM.

The area under the ROC curve for MAGE (0.618, 95%CI 0.555 to 0.680, p = 0.001) was superior to that for HbA_1c _(0.554, 95%CI 0.487 to 0.620, p = 0.129) (Figure 4).

## Discussion

Blood glucose level continuously fluctuates within a certain range in the human body. In diabetic patients, glycemic disorders include both sustained chronic hyperglycemia and glucose excursions. Patients with similar mean glucose or HbA_1c _levels can have markedly different glycemic excursions. Our study shows levels of FPG and HbA_1c _were not higher in CAD group patients than in the controls (p > 0.05). According to the traditional view, the two groups should have similar blood glucose control. However, we found that MAGE and PPGE levels in T2DM patients with CAD were higher than in T2DM patients without CAD. This result indicates that glucose excursions can not be neglected as an important aspect of glucose disorders and suggests it may be associated with coronary artery disease in T2DM patients.

Although the influence of glucose control, as assessed primarily by HbA_1c _levels, on the development of diabetes complications has been proven in numerous large-scale epidemiological studies [3,7,17], there is still an extensive debate about glucose variability as a risk factor for complications independent of HbA_1c _in diabetes[18,19]. A retrospective analysis from the Diabetes Control Complications Trial (DCCT) showed that HbA_1c_, as well as blood glucose fluctuation, seem to be associated with the microvascular complications of type1 diabetes [20]. A single study in T2DM demonstrated that fasting plasma glucose variability is a predictor of the onset of retinopathy in patients [21]. However, the controversial results were concluded from Kilpatrick et al. They independently performed analysis of the data of the DCCT showing that blood glucose variability was not related to the development or progression of either retinopathy or nephropathy in type1 diabetes [22]. Thirteen years later, the DCCT statisticians themselves corrected their previous findings and refuted the relation of glucose fluctuation and microvascular complications [23]. However, the DCCT was not designed to determine the impact of glycemic variability on the risk of the vascular complications of diabetes, and in fact the investigators required two attempts to report an acceptable statistical model. Since this was a post hoc analysis, at best it is hypothesis-generating.

In fact, more and more evidences have been found that glycemic variability may be an important parameter used to resolve potential clinical problems in diabetic patients [24]. Some researchers identified several important associations between postchallenge glucose excursions and known risk factors for atherosclerosis, and suggested that postchallenge glucose excursion is independently related to carotid intima-media thickness and may contribute to the development of atherosclerosis in individuals with T2DM independent of other risk factors [12,13]. The Verona Diabetes study reported that long-term variability of fasting glucose is an independent predictor of mortality in T2DM patients [25]. Some studies concluded that glucose variability was a significant predictor of mortality in critically ill patients independently from mean glucose level and severity of illness [26-28]. In the present study, MAGE≥3.4 mmol/L was found to be an independent predictor for the presence of CAD (OR 2.612; p = 0.002). Furthermore, the ROC plot also demonstrated that the MAGE level was a significant predictor for the presence of CAD (p = 0.001), whereas HbA_1c _was not (p = 0.129). These results indicate that intraday glucose excursion might contribute to generation of atherosclerosis even more specifically than sustained chronic hyperglycemia.

At present, the identified role of glucose variability in pathogenesis of atherosclerosis is not clear. Hyperglycemia is thought to induce oxidative stress and interfere with normal endothelial function by overproduction of reactive oxygen species, which results in atherosclerosis through several molecular mechanisms. In addition, glucose variability might contribute to these processes as well. Recent *in vitro *studies indicate that glucose fluctuations can activate nuclear factor-κB and protein kinase C (PKC) pathway, leading to a greater expression of the adhesion molecules and excess formation of advanced glycation end-products than stable high glucose [29,30]. Some studies reported that intermittent hyperglycemia induced a higher degree of apoptosis in endothelial cells than chronic hyperglycemia [10,31]. Quagliaro et al. showed that the apoptosis of endothelial cells exposed to intermittent high glucose may be related to a reactive oxygen species (ROS) overproduction, through PKC-dependent activation of nicotinamide adenine dinucleotide phosphate (NADPH)-oxidase [30]. NADPH oxidase is a major cause of atherosclerosis. That suggests blood glucose excursion plays an important role in the occurrence and acceleration of atherosclerosis in diabetes. However, the relationship between glycemic variability and oxidative stress was not consistently reproduced in human studies [11,32]. These discrepant findings might be explained by differences in duration and frequency of the periods with alternating glycemia as well as differences in methods used for oxidative stress quantification. Furthermore, severe glycemic disorders may adversely affect circadian variation of cardiac autonomic modulation and circadian blood pressure variability which are associated with mortality and morbidity of cardiovascular disease [33,34]. Takei et al. find acute hyperglycemia may induce sympathetic dysfunction through multiple mechanisms, including hyperglycemia, hyperinsulinemia, and increased oxidative stress, whereas coronary microvascular dysfunction is closely related to sympathetic activation [35].

In our study, Pearson correlation analysis showed that Gensini score correlated positively with the level of MAGE(r = 0.277, p < 0.001), PPGE (r = 0.167, p = 0.002) and HbA_1c_(r = 0.136, p = 0.011), indicating a pronounced proatherogenic effect of worse glycemic disorders, which is consistent with previous findings. Furthermore, multivariate regression analysis revealed that MAGE and HbA_1c _were independent risk factors for the severity of CAD. These results indicate that intraday glucose excursion is an important contributing factor in the severity of coronary artery disease, which is independent of the average level of blood glucose. Our results further indicate that current practice of relying mainly on HbA_1c _within a target range is inadequate for timely therapeutic adjustments and reducing the risk of coronary artery disease. To accomplish this goal, the CGMS appears to be a very useful tool in primary care and its use should be expanded for effective management of T2DM.

A few limitations of this study should be recognized. Firstly, the sample size was relatively small in this study, so that some subgroup comparisons may have lacked power to detect significant differences for selected variables. Secondly, although we had maintained the patients' anti-hyperglycemic therapy as usual and avoided glucose infusion during CGMS monitoring period, some factors, such as different diets, physical and emotional stress etc., which may affect levels of admission glucose fluctuations couldn't be all prevented. Thirdly, lack of microvascular complications data, we didn't include those risk factors in study. Fourthly, due to the fact that the present study was a cross-sectional design, our results only show the association between glycemic variability and prevalent CAD rather than incident CAD. All subjects in the present study were scheduled for coronary angiography for their suffering chest pain in cardiovascular department of Beijing Anzhen hospital. Therefore, it is likely that enrolled T2DM patients had greater risk for CAD than ordinary T2DM patients.

## Conclusions

The present study shows that the intraday glycemic variability is associated with the presence and severity of CAD in patients with T2DM. Recent accumulated evidence suggested that blood glucose excursions may play an important role in the occurrence and development of atherosclerosis in diabetes. There should be an emphasis on efforts to control all aspects of blood glucose disorders including HbA_1c_, FPG, postprandial glucose, as well as glucose fluctuations. Further well-designed studies are warranted to examine if reduction of glucose excursions has a substantial impact on CAD development in patients with T2DM.

## Competing interests

The authors declare that they have no competing interests.

## Authors' contributions

All authors listed on the manuscript participated in the design and coordination of the study and made substantial contribution to the intellectual content of the project to be included as authors. They also read and approved the final manuscript.
